# Mental Health Comorbidities in Pediatric Chronic Pain: A Narrative Review of Epidemiology, Models, Neurobiological Mechanisms and Treatment

**DOI:** 10.3390/children3040040

**Published:** 2016-12-02

**Authors:** Jillian Vinall, Maria Pavlova, Gordon J. G. Asmundson, Nivez Rasic, Melanie Noel

**Affiliations:** 1 Department of Anesthesia, University of Calgary, Calgary, AB T3B 6A8, Canada; jillian.vinall@ahs.ca (J.V.); nivez.rasic@ahs.ca (N.R.); 2 Department of Psychology, University of Calgary, Calgary, AB T2N 1N4, Canada; mpavlova@ucalgary.ca; 3 Department of Psychology, University of Regina, Regina, SK S4S 0A2, Canada; Gordon.Asmundson@uregina.ca; 4 Division of Behaviour and the Developing Brain, Alberta Children’s Hospital Research Institute, Calgary, AB T3B 6A8, Canada

**Keywords:** chronic pain, posttraumatic stress disorder, anxiety, depression, neurobiology, stress, brain, comorbidity, parent, intervention

## Abstract

Chronic pain during childhood and adolescence can lead to persistent pain problems and mental health disorders into adulthood. Posttraumatic stress disorders and depressive and anxiety disorders are mental health conditions that co-occur at high rates in both adolescent and adult samples, and are linked to heightened impairment and disability. Comorbid chronic pain and psychopathology has been explained by the presence of shared neurobiology and mutually maintaining cognitive-affective and behavioral factors that lead to the development and/or maintenance of both conditions. Particularly within the pediatric chronic pain population, these factors are embedded within the broader context of the parent–child relationship. In this review, we will explore the epidemiology of, and current working models explaining, these comorbidities. Particular emphasis will be made on shared neurobiological mechanisms, given that the majority of previous research to date has centered on cognitive, affective, and behavioral mechanisms. Parental contributions to co-occurring chronic pain and psychopathology in childhood and adolescence will be discussed. Moreover, we will review current treatment recommendations and future directions for both research and practice. We argue that the integration of biological and behavioral approaches will be critical to sufficiently address why these comorbidities exist and how they can best be targeted in treatment.

## 1. Introduction

Chronic pain, defined as pain occurring constantly or frequently for 3 months or more, is very prevalent in adolescence [1] and poses high costs to society ($11.8 billion/year) [2]. Poorly managed pain in childhood can lead to persistent pain problems and mental health disorders into adulthood [3,4]. In a longitudinal study, youth with chronic abdominal pain were found to be at greater risk of developing anxiety and depressive disorders as compared to youth without chronic pain [3,5]. Importantly, the risk of anxiety and depressive disorders were higher than controls regardless of whether or not chronic pain persisted into adulthood, suggesting that chronic pain in adolescence heightens the risk of developing mental health disorders, even when pain resolves [5]. Epidemiological research also suggests that youth with chronic pain have higher rates of anxiety disorders in adulthood [4]. This provides compelling evidence that youth with chronic pain are at risk for having and developing mental health conditions. Nevertheless, there remains limited understanding of the mechanisms associated with these comorbid mental health disorders that might impact children’s response to pain and pain treatment.

The co-occurrence of chronic pain and mental health conditions (i.e., posttraumatic stress disorder (PTSD)), anxiety and depressive disorders) is high, and has been explained by the presence of shared neurobiology (e.g., genes, hormones, brain networks) and mutually maintaining cognitive (e.g., attention and memory biases) and behavioral (e.g., sleep disturbance) factors that lead to the development and/or maintenance of both conditions [6,7,8]. However, existing interventions generally fail to effectively target co-morbid mental health disorders and underlying mechanisms that maintain both conditions [9]. It is becoming increasingly clear that there is a need for treatments of pediatric chronic pain to move away from the “one-size-fits-all” approach, which may not sufficiently help a substantial portion of youth with mental health co-morbidities. Indeed, treatment effects for psychological therapies for pediatric chronic pain have generally been small [10], which could be due to heterogeneity within pediatric samples in terms of these underlying mental health conditions. It has been suggested that by increasing our understanding of the genetic and neurobiological mechanisms underlying these comorbidities, we may be able to better predict trajectories of pain and tailor interventional strategies [11]. Moreover, particularly in youth, one cannot underestimate the interaction and influence of the environment (e.g., the parents’ influence) on children’s pain and mental health conditions.

This review will explore the epidemiology of mental health disorders in pediatric chronic pain populations, and present current working models that have been put forth to explain this comorbidity. Given that the vast majority of work on this topic has emphasized cognitive, affective, and behavioral mechanisms, we will highlight recent research investigating the shared neurobiological mechanisms that may underlie these comorbidities. Parental contributions to comorbid chronic pain and psychopathology in childhood will also be discussed. Finally, we will discuss current treatment recommendations and future directions for both research and practice. Given the high prevalence [1], large economic burden [12], and sometimes debilitating nature of pediatric chronic pain on physical and mental health [13] that can persist into adulthood [4], identifying and modifying mechanisms underlying these conditions has powerful implications for preventing adult-onset chronic pain and mental health disorders.

## 2. Epidemiology of Internalizing Mental Health Disorders in Pediatric Chronic Pain Population

### 2.1. Longitudinal Studies of Pediatric Chronic Pain and Internalizing Mental Health Issues in Adulthood

Evidence demonstrating the comorbidity of chronic pain and mental health disorders is accumulating (Table 1). One of the seminal epidemiological studies examining the co-occurrence of pediatric chronic pain and psychiatric disorders was undertaken by Hotopf et al. [5]. The 1946 birth cohort, followed for over two decades examined the relationship between chronic abdominal pain in childhood and medically unexplained symptoms, and mental health disorders in adulthood. They found that while the presence of pediatric abdominal pain was not associated with physical symptoms in adulthood, the risk of developing a psychiatric disorder by the age of 36 years was higher in children who had recurrent abdominal pain versus those who did not [5]. A similar study reported recurrent headaches in childhood to be related to an increased risk of psychiatric morbidity and multiple physical symptoms at the age of 33 years [3]. Despite the reliance on maternal and self-report, and failure to use a conventional definition of chronic pain (e.g., that included pain frequency in addition to pain duration), these studies laid an important foundation for the field of pediatric pain. They provided evidence that while pediatric chronic pain may or may not persist into adulthood, it confers risk of developing mental health disorders in adulthood.

Building upon these previous epidemiological studies, Walker et al. [16] conducted a longitudinal study of youth diagnosed with chronic abdominal pain followed into adulthood, at which time their pain and mental health status was assessed. The authors used cluster analysis to categorize youth with persistent abdominal pain into three groups based on pain ratings, gastrointestinal and non-gastrointestinal symptoms, beliefs about pain threat, pain coping efficacy, pain catastrophizing, negative affect and functional disability assessed in adolescence (i.e., Low Pain Adaptive, High Pain Adaptive, and High Pain Dysfunctional) [16]. Follow-up clinical interviews at the mean age of 21 years revealed that high pain ratings in conjunction with poorer coping skills and psychological functioning (i.e., characteristic of the High Pain Dysfunctional group) were associated with higher risk of developing functional gastrointestinal disorder (FGID) and non-abdominal chronic pain in adulthood [16]. The odds of having an anxiety or depressive disorder comorbid with FGID in adulthood were significantly higher only in the High Pain Dysfunctional group [16]. These findings provided further evidence of reciprocal relationships between pediatric chronic pain and mental health disorders. This study demonstrated for the first time that it is not pain per se but, rather, the combination of pain and psychological functioning that predicts the co-occurrence of pain and mental health disorders into adulthood. In a second investigation, Shelby et al. [4] examined the same cohort of youth with functional abdominal pain (FAP), as well as a pain-free controls whose mental health was assessed in adulthood. Fifty-one percent of youth with a childhood history of FAP developed an anxiety disorder over their lifetime, and 30% of youth had a current anxiety disorder at follow-up [4]. Depressive symptoms followed a different pattern; lifetime, but not current, risk of developing a depressive disorder was higher for individuals with chronic abdominal pain in childhood [4]. Risk of developing anxiety or depressive disorders was heightened for youth with chronic pain (versus controls) even when their pain resolved by adulthood. Temporal associations (based on retrospective recall) between mental health status and chronic pain suggested that anxiety disorders preceded the onset of chronic pain, whereas a depressive disorder followed it [4].

Recent data from a cohort study found that in a sample of 1420 youth, somatic complaints during childhood predicted generalized anxiety and depressive disorders at the age of 19–26 years [17]. Findings of the most recent epidemiological study by Noel et al. [18] revealed a consistent pattern. The study conducted a secondary data analysis of a sample of 14,790 individuals from the National Longitudinal Study of Adolescent to Adult Health to examine the co-occurrence of adolescent chronic pain and lifetime rates of mental health disorders [18]. In contrast to previous research, a broader range of chronic pain conditions (i.e., headache, abdominal and musculoskeletal pain) were assessed in adolescence, and lifetime history of mental health disorders (i.e., depressive and anxiety disorders) were subsequently assessed in adulthood. Individuals with, versus without, chronic pain in adolescence subsequently reported significantly higher lifetime rates of anxiety (21.1%) and depressive (24.5%) disorders [18]. After controlling for established confounding factors (e.g., age, sex, insufficient sleep, general health), chronic pain in adolescence was associated with a greater likelihood of having anxiety (odds ratio (OR) 1.33) and depressive (OR 1.38) disorders over the lifetime [18].

### 2.2. Co-Occurrence of Pediatric Chronic Pain and Internalizing Mental Health Issues

Other studies have focused on establishing concurrent rates of mental health disorders and chronic pain status in youth. Coffelt et al. [21] analyzed retrospective data from 3752 youth admitted with a primary diagnosis of chronic pain. Forty-four percent of adolescents had a comorbid mental health condition [21]. The diagnoses included mood disorders (28%), anxiety disorders (18%), conversion and somatization disorders (6%), and PTSD (2.4%) [21]. Simons et al. [23] also investigated the prevalence of anxiety symptoms in a large sample of youth with chronic pain. While only 11% of the participants reported clinically significant levels of total anxiety, 31% may have underreported their anxiety symptoms given their heightened scores on a social desirability scale [23]. Children with diffuse pain reported significantly higher levels of anxiety as compared to youth with musculoskeletal, abdominal or neuropathic pain [23]. Recently, Noel et al. [25] compared prevalence of PTSD symptoms in a sample of youth with chronic pain and their parents to pain-free peers. Findings revealed significantly higher levels of PTSD symptoms (including clinically significant symptoms) in youth with chronic pain and their parents [25].

Similar patterns of co-occurring pain and mental health disorders were found among youth with chronic headaches. In a large sample of youth, recurrent headaches were positively related to higher levels of anxiety and depressive symptoms across two broad age groups (12–14 years, OR 2.05; 15–17 years, OR 1.64) [20]. These results are consistent with those of a meta-analysis of mental health conditions in pediatric headache populations, which revealed higher levels of psychopathology symptoms in children and adolescents with headache as compared to healthy controls [19]. Tension-type headaches were associated with higher internalizing symptoms, which included anxiety and depressive symptoms, withdrawal and somatic complaints [19]. A meta-analysis of FAP revealed that levels of concurrent anxiety, depression and psychological distress were significantly higher in youth with FAP as compared to pain-free youth [26].

The co-occurrence of pediatric chronic pain and mental health disorders has been shown to differ by child sex [14,27]. In a representative sample of 3733 youth, abdominal pain and headaches were associated with co-occurring anxiety disorders in girls, but not boys [27]. Musculoskeletal pain exhibited a similar pattern, showing a strong association with anxiety disorders in girls, but not boys [27]. Conversely, boys were more likely than girls to develop externalizing mental health disorders comorbid with recurrent abdominal pain [14,27]. Both girls and boys were at risk for developing depression in the presence of musculoskeletal pains [27].

In addition to establishing prevalence rates of pediatric chronic pain and mental disorders, Tegethoff et al. [24] sought to determine the temporal primacy of each condition using structured clinical interviews assessing mental health disorders, parental reports of youth mental health disorders and adolescent self-reports of chronic pain in a sample of 6000 youth. More than a quarter of youth reported having both a mental health disorder and chronic pain in their lifetime [24]. Most youth who experienced any type of chronic pain (i.e., back/neck pain, headache, or other chronic pain) reported having an anxiety disorder (17.4%) and an affective disorder (10.06%) [24]. Having experienced any type of chronic pain increased the risk of developing anxiety (OR 2.42), eating (OR 2.63) and depressive (OR 2.32) disorders [24]. Temporal associations (albeit assessed via retrospective report) were reported between the onset of mental health disorders in childhood and the subsequent onset of chronic pain. There were no significant temporal associations between the preceding onset of chronic pain and the subsequent onset of psychological disorders later in life.

Taken together, the available literature demonstrates high levels of co-occurrence of pediatric chronic pain and mental health disorders and symptoms. A major limitation of much of the epidemiological work to date has been the suboptimal assessment of pain. Indeed, chronic or recurrent pain status has often been limited to a single binary question that fails to capture crucial aspects of chronic pain assessment, such as pain frequency, distress due to pain, and pain interference or disability. Moreover, over-reliance on retrospective self-report of symptoms has precluded reliable conclusions about risk and temporal relationships between the onset of chronic pain and mental health issues. The few studies examining temporality suggest that anxiety issues may indeed precede the development of chronic pain; however, rigorous prospective research is needed. Arguably the most striking finding from this body of research is that, irrespective of whether or not pediatric chronic pain resolves by adulthood, the long-term risk for developing mental health disorders remains. This has important clinical implications and suggests that treatments for chronic pain that only target pain, may not be sufficient for interrupting a trajectory of illness into adulthood.

## 3. Models of Chronic Pain and Comorbid Anxiety, Depression and PTSD

Several models have been proposed to understand the mechanisms underlying comorbidities between various mental health disorders and chronic pain in adults. Sharp and Harvey’s mutual maintenance model accounted for seven mechanisms through which PTSD and chronic pain might mutually maintain each other [8]. They proposed that cognitive (e.g., attentional biases), affective (e.g., depression) and behavioral (e.g., reduced activity levels) factors, characteristic of chronic pain, serve to exacerbate PTSD symptoms. Similarly, physiological (e.g., heightened alarm response to trauma reminder), affective (e.g., negative alterations on mood and cognitions) and behavioral (e.g., avoidance) aspects of PTSD contribute to the exacerbation of chronic pain [8]. The model, however, does not account for potential causal connections between those factors.

Liedl et al. [28] subsequently proposed the Perpetual Avoidance Model (PAM). The authors posited that trauma catalyzes dysfunctional cognitions and sensations of intrusion which lead to heightened arousal [28]. The role of hyperarousal is thought to be two-fold: it promotes avoidance behaviors that feed into PTSD-related dysfunctional cognitions and it amplifies pain sensations [28]. Intensified pain sensations further drive pain-related cognitions (e.g., catastrophizing, fear-avoidance beliefs), in turn, promoting avoidance of pain-inducing activities [28]. Avoidance (an element shared with PTSD) further intensifies pain sensations [28]. Hence, individuals get trapped in a cycle of perpetual avoidance that serves to maintain both PTSD and chronic pain. The PAM assumes no direct relationship between an inciting traumatic event and chronic pain. The pain model by Norman et al. [29] postulates that pain drives PTSD onset, whereas PTSD does not contribute to pain maintenance. Brown et al. [30] used structural equation modelling to test three theoretical models with a pediatric population (i.e., the PAM, mutual maintenance model, and the pain model). The authors used diagnostic interviews to assess PTSD and self-report to assess physical pain in a clinical sample of youth with traumatic brain injuries (TBIs) 3, 6 and 18 months after their injuries [30]. Sharp and Harvey’s model fit the acquired data significantly better as compared to the Norman et al. pain model. The PAM did not differ significantly in terms of fit as compared to Sharp and Harvey’s model, suggesting equally good fit [30]. The authors concluded that PTSD drives the presence of physical pain in this particular pediatric population.

Sharp and Harvey’s mutual maintenance model was later expanded to distinguish between mutual maintaining versus shared vulnerability factors, the latter of which serve to precipitate both conditions [7]. The shared vulnerability model postulates that certain psychological symptom clusters (e.g., anxiety sensitivity) and physiological factors (e.g., lower alarm threshold) can precipitate the emergence of both chronic pain and PTSD and be causally related to cognitive and behavioral mechanisms [6,7]. Moreover, the model posits that the conditions only develop when an individual with such a diathesis is exposed to a traumatic life event [7]. Trauma initiates a diathesis-amplified psychological response that includes maladaptive levels of fear and anxiety, and results in an array of detrimental behavioral (e.g., avoidance), cognitive (e.g., hypervigilance) and physiological (e.g., autonomic nervous system responsivity) consequences [7]. These consequences, which are thought to be bi-directionally related, account for the development of PTSD, chronic pain and/or both comorbidities [7]. The shared vulnerability model is the first model to include anxiety disorders, in addition to PTSD. Just as symptoms of mental health disorders (e.g., physiological arousal in anxiety, lack of positive emotions in anxiety or anhedonia in depression) may intensify pain symptoms [7], pain-related cognitive biases, physiological responses and maladaptive behaviors may serve to aggravate symptoms of mental health disorders [7]. It is, therefore, likely that mental health conditions and chronic pain share certain predisposing factors that form a common diathesis.

Until only recently, existing models of co-occurring mental health disorders and chronic pain were specific to adults. In a recent topical review, Holley et al. [31] proposed a new pediatric model of PTSD and chronic pain comorbidity in youth. Central to this model are shared vulnerability and mutually maintaining factors that are thought to influence PTSD, chronic pain and the disorders’ comorbidity in bi-directional ways. While many of the proposed factors are similar to those covered in the previous models (e.g., anxiety sensitivity, avoidance, hyperarousal), the authors drew evidence from pediatric research, confirming the important roles that these factors play in pain and mental health trajectories in childhood and adolescence [31]. Unique to this model is the integration of individual (e.g., trauma and pain-related variables), interpersonal (e.g., parent traumatic stress, peer victimization) and neurobiological (e.g., PTSD and pain influences on the developing brain) factors, all couched within a developmental context [31]. At all levels, PTSD and pain symptomatology are thought to reciprocally influence one another. For instance, catastrophic thoughts about pain in youth may lead to behavioral avoidance, and similar cognitions in parents may serve to exacerbate these cognitions and behaviors in their child [31]. Likewise, depressive symptoms characteristic of chronic pain and PTSD might be aggravated or even induced by peer difficulties and social isolation that are also commonly found in both conditions [31]. Overall, the proposed model fills a gap in the literature by being the first to integrate evidence from the pediatric PTSD and pain literatures, and positing developmentally relevant mechanisms that might underlie and serve to maintain both conditions in youth.

## 4. Potential Neurobiological Mechanisms Underlying Comorbid Chronic Pain and Anxiety, Depression or PTSD

As outlined in some of the models previously described (e.g., [6,7,31]), shared neurobiology of chronic pain and mental health conditions may in part explain the high co-occurrence of these disorders. Nevertheless, biological underpinnings of comorbidity have largely been overlooked relative to cognitive, affective and behavioral factors. There is accumulating evidence to suggest that genes, hormones and neural networks contribute to both the predisposition and maintenance of, chronic pain, PTSD, anxiety and depressive disorders. The following will provide a brief overview of some of the neurobiological factors that may underlie chronic pain and these associated mental health conditions. Presently, there are a limited number of studies that have examined mechanisms contributing to the development and maintenance of pediatric chronic pain and comorbid internalizing mental health issues. Therefore, in this particular section of the review, where there is an absence of child research, we draw from adult and animal literatures. We acknowledge that work in this area is preliminary. It is important that future research further investigate these factors and their developmental specificity to children and adolescents, so that interventions can be developed to effectively target the underlying mechanisms that maintain these conditions during the pediatric period.

### 4.1. The Hypothalamic–Pituitary–Adrenal Axis

Both stress and pain incite a cascade of neurobiological events, leading to the activation of the hypothalamic–pituitary–adrenal (HPA) axis and production of stress hormones (glucocorticoids (cortisol in humans)), which regulate the transcription of genes [32]. The process through which cortisol is secreted begins with the activation of the hypothalamus. This leads to the co-release of hormones [33,34,35,36] that stimulate the synthesis and release of adrenocorticotropin (ACTH) from the anterior pituitary gland. ACTH secretion influences the release of glucocorticoids from the adrenal cortex into general circulation [33,34,35,36]. Cortisol binds with glucocorticoid receptors in the hypothalamus, hippocampus and other brain regions to inhibit further production of cortisol (negative feedback) [33,34]. However, early and/or prolonged exposure to stress and/or pain can disrupt this cortisol feedback loop. Individuals with chronic pain often demonstrate HPA dysfunction [37,38], including altered glucocorticoid negative feedback [39,40] and abnormal cortisol levels [41,42,43,44]. Similarly, co-occurring stress-related mental health conditions (PTSD, anxiety and depressive disorders) are also associated with disruptions to the HPA axis [45,46,47].

Priming of the HPA axis begins in utero. Prenatal exposure to stress hormones leads to greater stress responses in adults, by reducing glucocorticoid receptors in the hippocampus [48,49]. Fewer glucocorticoid receptors in the hippocampus, leads to poorer negative feedback, and greater anxiety-related behavior during adulthood [48,49]. The postnatal environment also has considerable influence on the programming of the HPA axis. Natural variations in maternal behavior, such as the amount a rat licks and grooms their pups in the first week of life [50], incites a cascade of serotonin-mediated changes affecting hippocampal glucocorticoid receptor expression [51,52,53,54,55,56,57]. Adult offspring of low licking and grooming rat mothers showed reduced hippocampal glucocorticoid receptor expression, poorer glucocorticoid feedback sensitivity, and greater glucocorticoid production in comparison to pups reared by high licking and grooming mothers [52,53]. These changes in physiology are related to alterations in behavior as adults. Offspring of low licking and grooming mothers show greater anxiety-like behavior and alterations in cognitive functioning during adulthood [51,55,58,59,60], factors commonly associated with chronic pain [7]. Comparable disruption of cortisol reactivity in response to maternal behavior has been observed in humans. Early disruptions in the parent–child relationship have been associated with increased levels of cortisol in preschoolers [61]. Moreover, these heighted glucocorticoid responses were associated with increased behavioral and emotional problems both at school-age and adulthood [61,62]. Therefore, normal variations in early life experience and parent–child interactions can subsequently alter stress reactivity and behavior; this may contribute to the vulnerability and or mutual maintenance of psychopathology and chronic pain [7].

Cumulative life trauma also influences the development of chronic pain and psychopathology [63,64,65], which may, in part, be explained by dysregulation of the HPA axis. Youth with chronic pain report having a greater number of stressful life events compared to those without chronic pain, and cumulative trauma was associated with higher PTSD symptoms [25]. The timing and duration of traumatic events appears to largely influence glucocorticoid and behavioral responses to trauma. Both in humans and animals, prolonged pain and distress appears to be associated with a dampening of cortisol responses, in contrast to acute stressors that are associated with hyper-secretion of cortisol [52,53,66,67,68,69,70]. For example, among rhesus monkeys during the first month of life, when abuse is most prevalent, abused infants had elevated plasma cortisol levels as compared to non-abused infants [71]. However, by 6 months of age, the monkeys exhibited lower basal cortisol levels and attenuated ACTH to corticotropin-releasing factor (CRF) compared to control monkeys [71]. In humans, children who experienced abuse in their home environments, showed enhanced ACTH responses and normal cortisol levels [72]. However, adult survivors of childhood abuse or those with PTSD demonstrate lower ACTH responses following CRF injections [73]. Moreover, in a sample of youth exposed to interpersonal violence, if the trauma occurred in the last year of assessment, higher levels of salivary cortisol were positively associated with PTSD [74]. Conversely, in individuals with traumas exceeding a year prior to assessment, PTSD symptoms were associated lower levels of cortisol [74]. Ultimately, previous and/or prolonged physiological arousal associated with stress initiated by exposure to the threat of or actual death, serious injury, or violence can have detrimental effects on activation of neural and hormonal processes, and may contribute to the development of chronic pain and mental health disorders. This research also suggests that timing of exposure to traumatic/stressful events as well as assessment can influence the patterns of activation found.

Therefore, prolonged activation of the HPA axis may lead to stress-related conditions as chronic pain, PTSD, anxiety and depressive disorders. At this time, the mechanisms underlying the relationships between chronic pain and mental health conditions have not been well characterized. However, given the widespread actions of cortisol on multiple neurobiological systems, prolonged exposure to glucocorticoids may lead to changes in gene expression, the immune system and to the developing brain, thereby increasing the vulnerability and maintenance of these conditions.

### 4.2. Serotonin

Serotonin (5-hydroxytryptamine (5-HT)) is a widely distributed neurotransmitter that is a key modulator of stress responses (i.e., HPA axis) [75,76]. It is also an important molecule for pain processing, and is centrally involved in chronic pain states [77,78]. Previous studies have shown that individuals with chronic pain have decreased levels of 5-HT in their serum and cerebrospinal fluid [79,80]. This led to an investigation as to whether variants of 5-HT genes were associated with increased risk of developing chronic pain. One of the major 5HT receptor subtypes is the serotonin 2A receptor (5-HT2AR). It was found that following nerve injury, 5-HT2AR promotes spinal hyper-excitation and impairs spinal μ-opioid mechanisms, thereby contributing to the development of mechanical allodynia [81]. Using 2 population-based cohorts, Nicholl et al. [82] were able to demonstrate for the first time that single-nucleotide polymorphisms (SNP) of the 5-HT2AR gene were associated with musculoskeletal pain in adult males, even after adjustment for symptoms of depressive disorders. Therefore, 5-HT availability appears to have a role in the development of chronic pain conditions. Central to the reuptake of 5-HT is the transporter protein (5-HTT). Genetic variants of the transporter promoter region (5-HTTLPR) play a critical role in determining intra-synaptic 5-HT signaling [83]. In humans it has been found that the short (S) allele of 5-HTTLPR results in ~50% reduction in 5-HT reuptake compared to the long (L) allele [84]. The S allele has been found to increase the risk of chronic pain [85,86]. Chronic pain patients with the S allele also demonstrated higher levels of anxiety and depressive disorders [85]. It has been theorized that the inability for rapid 5-HT clearance from the synaptic cleft may result in serotonin 1A receptor (5-HT1A) subtype negative feedback, causing an overall decreased in 5-HT neurotransmission for individuals with the S allele [85]. Converging evidence from both human and nonhuman primates suggests that S allele carriers may also have greater vulnerability for anxiety and depressive disorders [87]. Indeed, a recent meta-analysis found a significant association between 5-HTTLPR genotype and HPA-axis reactivity to acute psychosocial stress, with homozygous carriers of the S allele displaying increased stress hormone reactivity compared with individuals with the S/L and L/L genotype [88]. The 5-HTTLPR genotype also modulates brain activation during emotional processing tasks [89,90]. Human adult functional magnetic resonance imaging (fMRI) studies have found consistent associations between the short allele and greater amygdala reactivity to aversive versus neutral stimuli [91,92]. This is important given that the amygdala plays a key role in activating the HPA axis. Altogether, it would appear that 5-HT availability modifies brain responses and predisposes individuals to developing chronic pain and/or mental health conditions.

### 4.3. Brain-Derived Neurotrophic Factor

Serotonin interacts closely with brain-derived neurotrophic factor (BDNF), a neurotrophin that is widely expressed in stress-related brain regions (e.g., prefrontal cortex and hippocampus) and is intrinsic to neurogenesis and synaptogenesis [93]. Both human and animal studies have demonstrated that stress hormones modify BDNF expression, such that there is decreased BDNF expression in the hippocampus and increased expression in the amygdala [94,95,96]. This finding is consistent with recent neuroimaging studies, which have reported significant loss in hippocampal neuroconnectivity with the medial prefrontal cortex [97], and exaggerated amygdalar connectivity with the central executive network (dorsolateral prefrontal cortex and posterior parietal cortex) in individuals with chronic pain compared to healthy controls [98]. Therefore, genetically determined BDNF availability may modify brain connections contributing to the development and maintenance of chronic pain conditions. To that effect, it has been shown that induced chronic inflammatory pain dampens hippocampal *BDNF* gene expression in rats [99]. Deletion of *BDNF* also produces chronic pain and depressive-like behavior in mice [100], and stimulators of BDNF synthesis have been shown to have an analgesic effect and reduce depression-like behavior in rats with chronic pain [101]. Similar to 5-HT, there are variants of *BDNF*, which are important determinants of intracellular processing and secretion [102]. Unlike Val66Met (rs6265), the 66Met allele results in lower BDNF availability, and is associated with alterations of human hippocampal function [103]. Both human and mouse 66Met allele carriers have been shown to have smaller bilateral hippocampi, in addition to lower gray mater volumes (e.g., prefrontal cortex), and reduced white matter tract integrity when compared to 66Val homozygote controls [104,105,106,107,108,109,110]. Furthermore, adult human and rat studies have shown that 66Met carriers may be at higher risk for developing chronic pain, PTSD, anxiety and depressive disorders [111,112,113,114,115,116,117,118,119]. Therefore, stress-regulated *BDNF* may be another mechanism through which genes may increase susceptibility to chronic pain and comorbid mental health conditions.

### 4.4. Inflammation

Another important factor to consider in the co-occurrence of chronic pain and mental health disorders is the effect that chronic pain and stress can have on immune function. Injury leads to the activation of microglia in the dorsal horn of the spinal cord, which results in the release of cytokines and growth factors that excite nociceptive dorsal horn neurons, contributing to the development of central sensitization and hyperalgesia [120] as well as the pathogenesis of chronic pain [121,122]. It is important to note that chronic inflammatory processes are not specific to pain (e.g., autoimmune diseases, aging) [123,124] and may arise from acute immune challenges [125]. Therefore, other conditions that incite neuro-inflammatory responses may also lead to chronic pain and comorbid conditions. Post-mortem immunohistochemical studies of the spinal cord in patients with complex regional pain syndrome [126] and human immunodeficiency virus-related neuropathic pain [127], and cerebral spinal fluid sampling of patients with fibromyalgia and chronic low back pain [128,129], have provided support for the involvement of glia in the pathogenesis of chronic pain. A recent study using positron emission tomography–magnetic resonance imaging showed for the first time, in vivo, that the occurrence of glial activation, as measured by an increase in [^11^C]PBR28 binding, was greater in the thalamus of patients with chronic pain as compared to controls [130]. Preclinical research involving nerve injury models have shown that chronic pain evokes anatomically specific neuroinflammation in brain regions that is causally linked to anxiety and depressive-like symptoms [131]. Specifically, nerve injury elicits the production of pro-inflammatory cytokines both in the periphery and centrally, which can cause a reduction in BDNF (neurogenesis) and glucocorticoid receptor expression and function, and an increase in excitoxicity in brain regions that are critical for behavior regulation (i.e., hippocampus, hypothalamus, amygdala, prefrontal cortex) [131,132,133,134,135]. Altering the connectivity of these regions can lead to alterations in function and behavioral disturbances. In humans, the production capacity of several cytokines has been positively associated with severity of depressive and anxiety symptoms, even while taking lifestyle and health factors into account [136]. Furthermore, it has been shown that among chronic pain patients, higher levels of tumor necrosis factor-α (TNF-α) and interleukin-6 (IL-6) were associated with less improvement in pain intensity, greater psychological inflexibility and lower mental health-related quality of life following a pain intervention, compared to patients with lower levels of these cytokines [137]. Therefore, inflammation may not only act as a precipitating factor for pain and mental health conditions, but also may be a perpetuating factor that deters patient recovery.

To date, very little research has been completed examining inflammatory factors and its contribution to the chronicity of pediatric pain and comorbid mental health disorders. Therefore, this represents an important, cutting-edge area for future research. We know that early life pain and/or stress also appears to dysregulate the developing immune system. Rat pups surgically incised on postnatal day 3 show priming of the central neuroimmune response, such that upon re-injury during adulthood, they demonstrate an enhanced degree and duration of microglial reactivity [138]. This is one of the first studies to demonstrate how early tissue injury can modify the neuroimmune profile to shape nociceptive processing throughout life, and may yield valuable insights into the potential link between pediatric and adult chronic pain conditions [138]. To the best of our knowledge, this has not yet been demonstrated in humans. However, Mitchell and Goldstein [139] reviewed 67 studies including nearly 4000 youth with mental health conditions and found preliminary evidence for elevated markers of inflammation in this population. In particular, associations with depressive disorders and PTSD converge with the extant adult literature demonstrating associations with inflammatory markers [139]. Early-life stress leads to a suppression of inflammatory markers during development, but causes a shift towards a pro-inflammatory state in later life [140]. For children born extremely preterm, this change in inflammatory responses may coincide with the dampening of HPA activity over time [69,70]. Prolonged stress appears to have a similar effect on inflammatory responses, such that in adult patients with comorbid PTSD and major depressive disorder demonstrated higher IL-6 activity concurrent with reduced sensitivity to glucocorticoids as compared to PTSD patients alone [141]. Furthermore, another study found that combat veterans with PTSD demonstrated abnormally elevated neuroinflammatory responses to deep pain stimuli relative to combat veterans without PTSD [142]. Taken together, inflammatory responses appear to play an important role in the development and maintenance of comorbid chronic pain and mental health disorders. Currently, many new pharmacological agents that target cytokines are being synthesized for different clinical indicators [143], which may result in better control of chronic inflammation, as well as improved treatment outcomes for patients with chronic pain and PTSD, anxiety and depressive disorder comorbidities.

### 4.5. Neuroimaging Chronic Pain and Psychopathology

In recent years, several neuroimaging studies have attempted to characterize nociceptive systems, their central nervous system targets (e.g., primary and secondary somatosensory cortices, insula, anterior cingulate cortex, and thalamus) [144,145,146,147,148], and correlate these regions with the modality and intensity of noxious input [144,149,150,151]. For example, in a recent fMRI study involving 114 healthy adult participants, Wager et al. [151] found a highly sensitive and specific neurologic signature of physical pain. It included both medial (e.g., affective; anterior cingulate cortex) and lateral (e.g., sensory; somatosensory cortices) pain systems that were consistent across individuals [151]. However, in the transition from an acute and adaptive pain state to a chronic, maladaptive neuropathic disease state, there is extensive functional and metabolic reorganization in pain-related brain circuitry. A recent study by Hubbard et al. [152] examined functional brain changes over time in response to acetone application to the left hindpaw in rats that either received a spared nerve injury (SNI) or sham surgery. The SNI rats demonstrated early hyperactivity of sensory areas (ventroposterior lateral nucleus of the thalamus, primary somatosensory cortex) and later hyperactivity of affective areas (anterior cingulate cortex, prelimbic cortex), and early and sustained hypoactivity of the medial thalamus and periaqueductal gray matter [152]. Moreover, for the SNI rats, these functional brain changes were associated with early and sustained increases in behavioral measures of mechanical and cold sensitivity [152]. Therefore, it would appear that as an individual transitions from an acute to chronic pain state, activations within the brain move from primarily somatosensory regions to limbic regions, indicating a shift from primarily physical to emotional neural processing. These findings were recently corroborated by Jensen et al. [153]. Using an activation likelihood estimate to analyze 138 independent data sets, they demonstrated that chronic pain patients were less likely to activate key nociceptive regions compared to healthy controls [153]. In low back pain patients followed longitudinally, Mutso et al. [97] showed that over the course of 1 year, patients acquired significant losses in hippocampal connectivity with the medial prefrontal cortex. This cellular loss between the hippocampus and cortex seemed to contribute to the transition from subacute to chronic pain and was thought to be a factor in the known aversive learning and heightened anxiety associated with chronic pain. Baliki et al. [154] also noted over the same time course that gray matter brain density decreased in patients with low back pain relative to healthy controls. However, adult chronic low back pain patients also tended to have exaggerated amygdalar connectivity with the central executive network (dorsolateral prefrontal cortex and posterior parietal cortex) as compared to healthy controls [98]. Moreover, this greater connectivity has been associated with increased tendencies to engage in catastrophic thinking about pain [98]. In support of this finding, in adolescents with irritable bowel syndrome, disease duration is associated with cortical thickness in bilateral dorsolateral prefrontal cortex and left supramarginal gyri [155]. Additionally, higher levels of pain intensity were associated with significant cortical thickening in the bilateral orbitofrontal cortex [155]. Thus, similar to the animal research described above, in humans, the transition from acute to chronic pain is characterized by reorganization within the limbic structures, thereby altering cognitive and emotional processes. This also supports the potential role of cognitive (e.g., memory and attentional biases) and affective (e.g., depressive symptoms) factors as potential mechanisms that may maintain the comorbidity between chronic pain and mental health conditions.

To date, very few neuroimaging studies have been conducted in pediatric chronic pain populations. However, among the few pediatric studies, similar findings to studies in adults have been reported. Youth with complex regional pain syndrome have been shown to have greater connectivity of the left and right amygdala to several cortical and subcortical areas as compared to healthy controls [156]. However, despite this greater connectivity, Simons et al. [157] found decreased evoked responses to fearful stimuli in patients with complex regional pain syndrome compared to healthy sex- and age-matched controls in prefrontal and limbic regions, particularly within the striatum, amygdala, insula and dorsolateral prefrontal cortex. Furthermore, blunted responses to fearful expressions in the caudate, putamen, centromedial amygdala, and anterior insula were associated with pain-related fear levels. Simons et al. [157] postulated that these results corroborate accumulating research demonstrating alterations in cognitive-affective brain regions in the chronic pain state [158] and may reflect either allostatic over-load [159,160] or pain avoidance, a maladaptive behavior often used in an effort to manage chronic pain [157].

Although the majority of neuroimaging studies of pediatric chronic pain have focused on the amygdala and its functional connections, pain is a multidimensional experience, with sensory, cognitive, and evaluative aspects. Therefore, other networks may be simultaneously activated during the transmission of pain signals in the brain. Differences in intrinsic brain networks were observed in pediatric complex regional pain syndrome patients as compared to controls, with the most prominent differences in the central executive, default mode, sensorimotor, and salience (anterior insula, mid-cingulate cortex, temperoparietal junction, and dorsolateral prefrontal cortex) networks [161]. Given the extent of neural networks that are affected by chronic pain, it is not surprising that comorbidities (i.e., PTSD, anxiety and depressive disorders) exist among individuals with chronic pain. Indeed, these same networks have also been shown to be altered in individuals with PTSD, anxiety and depressive disorders [162,163,164,165]. The majority of neuroimaging studies to date have only examined one to two of these disorders in conjunction with pain and their effects on the brain. Therefore, the specificity of the overlap within the brain between each of these disorders requires further investigation. However, it is becoming increasingly clear that chronic pain, anxiety, depression and/or PTSD, are not completely independent and the predisposing factors and neurobiological mechanisms, which lead to the presence and/or comorbidity of these disorders, are intrinsically related.

## 5. Parental Mental Health Disorders in Pediatric Chronic Pain Population

As previously mentioned, the neurobiology of pediatric chronic pain is deeply embedded in the broader social context of the family and parent–child relationship. It is, therefore, essential to examine parental factors that co-occur with pediatric pain or that might contribute to the development of chronic pain and comorbid mental health conditions. Several studies and reviews have confirmed a strong relationship between familial history of chronic pain with pediatric chronic pain [166,167]. Hoftun et al. [168] used a large community sample of Norwegian youth and at least one of their parents to examine the relationship between parental chronic pain history and pediatric chronic pain. The youth were more likely to have chronic pain if their mother or father reported recurrent pain issues; the risk was much higher if both parents were suffering from chronic pain [168].

There is evidence to suggest that genes, in combination with early environments, contribute to the risk of developing chronic pain and comorbid mental health conditions. Parents with the *5-HTTLPR* S allele exhibit significantly less observed positive parenting than those with the L genotype [169]. The S allele results in ~50% reduction in 5-HT reuptake compared to the long (L) allele [84], which is thought to cause an overall decrease of 5-HT neurotransmission in individuals with the short *5-HTTLPR* allele [85]. Lower 5-HT availability, leads to reduced glucocorticoid receptor expression, and greater circulating cortisol [51,52,53,54,55,56,57]. As previously described, children of mothers that have increased stress hormones during pregnancy are more likely to have greater stress responses as adults [48,49]. However, the postnatal environment is also important for HPA axis programming, such that parent behaviors can either reduce or increase cortisol activity, thereby modifying gene expression [52,53,170,171]. Glucocorticoid dysfunction is associated with chronic pain and mental health conditions (PTSD, anxiety and depressive disorders) [45,46,47]. Therefore, parent factors contribute to their child’s risk of subsequently developing chronic pain and comorbid mental health conditions.

Other studies have also reported associations between parental mental health and chronic pain in children. Garber et al. [167] examined children with recurrent abdominal pain (RAP) with and without known “organic cause”, children with psychiatric issues and a healthy control group. Parental self-reports of psychiatric symptoms were significantly different between the groups. Mothers (but not fathers) of children with RAP were more anxious than mothers of children who had pain with a known “organic” origin and mothers in the control group [167]. Mothers of children who had RAP without a known “organic cause” reported more depressive and somatization symptoms than mothers of healthy controls. Walker et al. [172] reported similar findings: mothers of children with RAP reported higher levels of depressive, anxiety, and somatization symptoms as compared to mothers of healthy children. Parental reports did not differ significantly between the groups. Campo et al. [173] examined a clinical sample of children with FAP and their mothers, finding the latter were significantly more likely to report a lifetime incidence of anxiety or depressive disorders as compared to mothers of children without recurrent pain. The UK child development cohort study mirrored those findings: high levels of maternal neuroticism was a risk factor for pediatric abdominal pain [5], and family history of mental health issues were linked to recurrent headaches in childhood [3,21]. Williamson et al. [174] examined a small clinical sample of youth with chronic pain and their mothers. In addition to child ratings of pain, another significant predictor of child-reported depression was maternal depression. Similarly, Wolff et al. [175] assessed anxiety and depressive symptoms in pregnant women as a part of a population-based cohort study (the Generation R). Among other factors (e.g., maternal stress and child temperament), higher anxiety symptoms in mothers increased the risk of somatic complaints in their 18 month old children [175]. While the number of studies in this area is rather limited, a pattern of high co-occurrence between pediatric chronic pain and parental mental health disorders and symptoms is evident.

Moreover, as reported in a recent meta-analysis, children of parents who have versus who do not have chronic pain are more likely to develop a host of physical and mental health problems from early in life [176]. Starting as early as perinatal period, children of mothers with chronic pain were at higher risk for preterm delivery, C-section, and adverse birth outcomes (e.g., low birth weight, congenital abnormalities) [176]. In addition to increased pain problems, offspring of parents with chronic pain were at higher risk for poorer mental health outcomes [176]. Limited evidence was found for higher rates of externalizing problems [176]; however, consistent patterns were found for internalizing problems. Levels of both parent- and youth-reported internalizing problems (including symptoms of depression, anxiety and obsessive–compulsive disorder) in youth whose parents had chronic pain were higher as compared to controls (i.e., children of pain-free parents) [176]. Other research has examined parent–child transmission of gastrointestinal illness (GI) behaviors and psychosocial mechanisms underlying this transmission [177,178]. In a study of parents with and without irritable bowel syndrome and their children, a stronger relationship between maternal and child psychological distress was found among the clinical group [178].

Stone and Wilson [179] recently developed an integrative conceptual model addressing the apparent clustering of chronic pain within families. The model outlines possible mechanisms (e.g., pain-specific social learning, general parenting style) and child vulnerabilities (e.g., altered pain processing, pain-related cognitions, pain coping behaviors, physical health, emotion regulation) that contribute to intergenerational transmission of chronic pain risk and may lead to adverse pain-related child outcomes (chronic pain, poor psychological functioning, disability) [179]. The authors also identified potential moderators that exacerbate the risk of transmitting chronic pain to offspring. Thus, chronic pain status of the second parent increases the risk of child developing chronic pain, whereas a pain-free second parent may buffer this risk [179]. Earlier and longer exposure to parental chronic pain, that is, in the same location as child pain is more detrimental as it increases the chances of child learning and adopting pain-related behaviors and beliefs [179]. Girls, African American youth and youth exposed to parental chronic pain at earlier ages may be at higher risk for developing chronic pain as compared to boys, non-Hispanic White youth, and youth who were exposed to parental chronic pain during late adolescence [179]. Importantly, it was proposed that positive affectivity in the child might buffer against the negative influence of parental chronic pain on children, whereas negative affectivity and attention control (i.e., effortful control) in the child may have the opposite effect [179].

Many of the same mechanisms that have been proposed to underlie parental and child chronic pain may also underlie the familial transmission of chronic pain and internalizing mental health symptoms between parents and youth. These proposed mechanisms include: (1) genetic contributions; (2) modeling of behavior (i.e., mothers with higher levels of anxiety model associated patterns of behavior to their children) and pain-specific learning; (3) changes in parenting style, behavior, and interactions with the child (e.g., withdrawal associated with depression, excessive attention to child’s pain in cases of heightened anxiety); (4) early neurobiological changes; (5) general familial health; and (6) exposure to stressful environment [167,179]. However, it remains unclear whether parental mental health is a risk factor for development of pediatric pain or a result of having a child with a pain problem. Certainly, parenting a child with chronic pain is associated with high emotional burden to the parent and the broader family system, and recent efforts have been made to directly target parental distress in pediatric chronic pain for this very reason [180]. Twenty percent of parents of youth with chronic pain (versus 1% of parents of pain free peers) have been found to experience clinically significant levels of PTSD [25], however, their PTSD symptoms were not related to those of their child [25].

Any temporal and/or causal connections between pediatric chronic pain and parental psychological health are premature and require further longitudinal and epidemiological investigations. It will also be important to consider that parental mental health might be comorbid and have a bi-directional influence on youth’s psychological functioning in chronic pain populations. Palermo et al. [181] developed a conceptual three-level model that elucidates factors at play in pediatric chronic pain. Parental level variables (mental health and emotions, behaviors, physical health status) connect to the other two levels including family variables and developmental factors, by the means of bi-directional relationships. Clearly there is a necessity to further investigate parental factors to gain a more comprehensive understanding of their role in the onset and trajectories of pediatric chronic pain and mental health.

## 6. Treatments for Comorbid Chronic Pain in Youth

Despite growing recognition of the high co-occurrence of mental health disorders in children and adolescents with chronic pain, treatment approaches to address this growing problem have lagged behind. Currently, psychological treatments for chronic pain have taken a “one-size-fits-all” approach and predominantly focus on changing pain-related cognitions and behaviors without adequately addressing elevations in anxiety and depressive symptoms that often accompany the pain. This could, in part, explain the small and non-significant treatment effects that have been found for face-to-face psychological interventions in reducing pain and disability among headache and non-headache pain populations, respectively. While it may also account for a lack of lasting reductions found in anxiety and depressive symptoms following treatment, this could also reflect the small number of trials that have analyzed mental health conditions as outcomes [10]. Similarly, in a recent meta-analysis of remotely delivered psychological interventions (e.g., via the internet, computers, smartphone applications or telephone), there were no beneficial effects found in reducing depressive symptoms and only small effects in reducing headache pain in the short-term [182]. However, it should be noted that there were only a small number of (small) trials conducted, and, like the face-to-face trials, many did not assess both anxiety and depressive symptoms as primary outcomes. Taken together, the evidence to date suggests that our existing interventions for chronic pain emphasize changing pain cognitions and behaviors without adequate emphasis on co-occurring mental health conditions. Although more trials assessing pain and mental health disorders as primary outcomes are needed, it is possible that existing treatments may not be sufficient for interrupting a trajectory of pain and comorbid mental health conditions from persisting long-term.

Recent research suggests that mental health comorbidities and symptoms can impede recovery among youth with chronic pain. Cunningham et al. [183] examined 175 children seen in a pediatric pain management center, 40 of who completed cognitive–behavioral therapy for chronic pain. Findings revealed that children who had clinically significant symptoms of anxiety at the time of initial evaluation (albeit similar levels of pain and functioning) showed greater initiation in and completion of the pain intervention but poorer treatment response (i.e., less reductions in pain intensity and functional disability) as compared to youth with subclinical anxiety symptoms. Research also suggests that modifying anxiety is important for reducing pain and improving functioning during intensive interdisciplinary rehabilitation pain programs. Specifically, Benore et al. [184] found that over and above the effect of age, sex and pain at admission and 1-month post-discharge, reductions in general anxiety, pain-specific anxiety and pain catastrophizing predicted improved physical quality of life, as well as pain intensity, but, not the number of missed school days. Taken together, this evidence demonstrates that mental health (anxiety) symptom elevations impede recovery in psychological pain treatments and should be reduced in order to see improvements in pain and functional outcomes. This suggests that youth with chronic pain are not a homogenous group and treatment approaches that are tailored to addressing co-morbid anxiety symptoms are likely necessary for subgroups of youth who exhibit clinically significant anxiety symptoms. Moreover, youth who appear to be most engaged in treatment (i.e., those with clinically significant anxiety symptoms) might be the very ones who are least likely to recover. Research on treatment response among youth with other mental health conditions (e.g., depression, PTSD) is needed.

Like Cunningham et al. [183], recent authors have argued for the use of mental health screening tools to identify mental health disorder co-morbidities [31,185,186] in order to predict outcomes and inform clinician-use of treatment approaches. Some authors have also provided treatment recommendations [31,185], albeit largely based on adult research. Adult guidelines recommend treating overlapping pain and PTSD symptoms (e.g., fears and avoidance behaviors) concurrently [9,187]. Dually targeted interventions have been developed for adults that combine PTSD behavioral activation, pain education and emphasis on exercise [9,188]. Similarly, other programs developed for adult veterans have combined cognitive processing therapy for PTSD with CBT for chronic pain [9,189]. Pediatric researchers have stressed the importance of understanding comorbid PTSD and chronic pain within a developmental context [31] and cautioned against simply downwardly extending adult recommendations to pediatric populations. Attempts have been made to apply a unified, transdiagnostic treatment for adolescents with chronic pain and comorbid anxiety and depressive disorders (Unified Protocol for the Treatment of Emotions in Youth With Pain (UP-YP)) [190]. The UP-YP is a modular-based individual treatment protocol [190]. Treatment is offered between 8 to 21 50-min sessions over a front-loaded 6-month period [190]. The UP-YP modules include: (a) psychoeducation about emotions and pain; (b) awareness of emotions and pain; (c) flexibility in thinking; (d) modifying emotion-driven behaviors through exposures; and (e) treatment review and relapse prevention [190]. Several optional modules are available as well (e.g., parenting the emotional adolescent with pain) [190]. Although similar in its application to traditional CBT therapy, the UP-YP aims to modify both pain and emotions [190]. Using two case studies, Allen et al. [190] provided initial evidence that the UP-YP can be used to broaden the scope of treatment to include both emotion regulation skills and cognitive–behavioral treatment strategies to assist individuals with complex medical and psychological conditions. After completing the training modules, case 1 was back at school nearly full-time, engaging in social activities, and showed significant improvement in his anxiety and disability, although his pain levels remained more or less the same. Case 2, was somewhat less successful as her pain symptoms were initially being addressed by the program, but her physical illness towards the end of treatment precluded her from the continued benefits of UP-YP. As an alternative to this method of intervention, clinicians may choose to stagger treatment delivery by treating the condition that causes the most distress first and then addressing remaining symptoms of the other condition; however, treatment algorithms based on evidence are needed. Clearly, knowing when and how to integrate versus stagger treatment is a critical area for future research in this area.

Another reason why existing interventions may not help a substantial portion of youth may be due to the fact that they do not effectively target the underlying mechanisms that maintain these conditions. Genetic screening for alleles associated with these conditions may help to identify patients at risk for developing chronic pain and a comorbid mental health condition, allowing clinicians to intervene prior to significant neural remodeling. A better understanding of the neural “signature(s)” associated with chronic pain, PTSD, anxiety and depressive disorders will also help to evaluate the effectiveness of clinical interventions for altering this neural circuitry. New therapies, which directly target the underlying mechanisms of these conditions, may also be a promising new direction for clinical intervention. For example, transcranial magnetic stimulation (rTMS) involves using a magnetic field generator to stimulate small regions of the affected brain region. Thus far, rTMS has been shown to be an effective treatment for burning mouth syndrome [191], complex regional pain syndrome [192], fibromyalgia [193,194], neuropathic pain [195,196,197], depressive disorders [198] and to a lesser degree PTSD and other anxiety disorders [199,200]. It has been proposed to prevent or revert the ongoing maladaptive plasticity within the pain matrix [201], and modify opioidergic, glutamatergic, gamma-aminobutyric acidergic, and serotoninergic neurotransmissions [202]. The application of screening, implementation of early interventions at the individual and familial level (e.g., teaching coping mechanisms, parent sensitivity training), refining current intervention strategies, and the innovation of new, targeted therapies, holds promise for addressing and reducing these comorbidities.

## 7. Discussion

**Figure 1:**
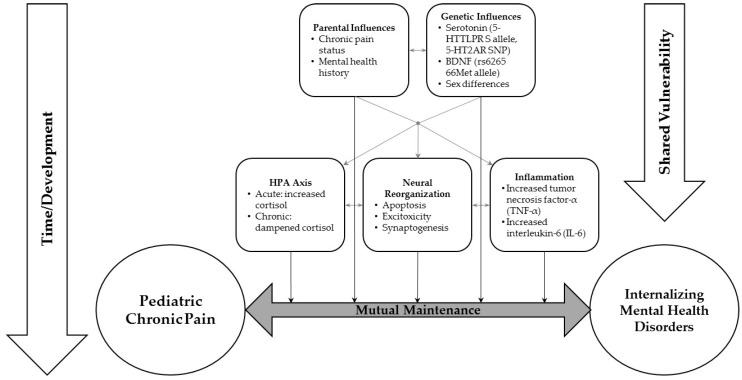
Possible mechanisms underlying the shared vulnerability and mutual maintenance of pediatric chronic pain and internalizing mental health disorders. 5-HTTLPR: Serotonin-transporter-linked polymorphic region; BDNF: Brain-derived neurotrophic factor; HPA: hypothalamic–pituitary–adrenal; S allele: Short allele.

High levels of comorbid chronic pain and mental health conditions have been reported in pediatric populations, and persistent pain reportedly increases the risk of PTSD, anxiety and depressive disorders later in adulthood [3,4,5,16,17]. Chronic pain and mental health disorders (as well as their subclinical symptoms) reciprocally influence each other, significantly decrease youth quality of life and impose a large health care and economic burden in childhood and adulthood [2,7,18,31]. While studies have attempted to identify mechanisms underlying these relationships, they often focus on one to two factors, which cannot account for the complex interplay between genetic, epigenetic, chemical, neuronal and environmental factors that may interact synergistically to influence the development and maintenance of chronic pain and mental health conditions. This review highlights some of the ways in which pre-determined and concurrent neurobiology and genetics interact with parental influences to influence chronic pain and mental health conditions in youth (Figure 1). It extends previous pediatric and adult work on mental health conditions/symptoms and chronic pain, which have focused primarily on cognitive, affective, and behavioral mechanisms. We suggest that an integrative approach combining biological and behavioral factors is needed to push forward this important line of inquiry, and ultimately inform how to interrupt this trajectory, to prevent issues from persisting into adulthood. However, systematic review methodology is warranted as more evidence accumulates on this topic.

Theoretical work is critical for synthesizing existing (and often disparate) bodies of evidence and guiding future scientific inquiry. Existing models that address comorbidity of mental health disorders and chronic pain are scarce. Moreover, they focus primarily on the relationship between PTSD and chronic pain, and are primarily based on adult populations [7,8,28]. While mutual maintenance [8] and shared vulnerability [6] models account for the relationship between PTSD and persistent pain in adults, they do not account for an array of dynamic changes that occur during childhood and adolescence and the unique influence of parents. The new developmentally informed model of Holley et al. [31] begins to fill this gap by placing shared PTSD and chronic pain factors within interpersonal (e.g., parent context) and neurobiological (e.g., impact of pain and PTSD on the developing brain) developmental contexts. A more generalized model addressing the comorbidity between several mental health disorders/symptoms (depression, anxiety and PTSD) and chronic pain in youth would help guide future research and inform clinical practice in this area.

There has been extensive literature examining the contribution of specific genes and neurobiological pathways underlying chronic pain, anxiety, depression and PTSD in adults and animals. However, these studies are often conducted in isolation of one another and focus specifically on the neurobiological influence on one or two outcomes in particular. We have demonstrated in this review that many of the same genes (e.g., 5-HT, *BDNF*), hormones (e.g., cortisol), neurotransmitters (e.g., 5-HT), cytokines (e.g., TNF-α, IL-6) have been implicated in each of these conditions and are inextricably related to one another, either directly or indirectly through the HPA axis. For example, individuals with the short (S) allele of 5-HTTLPR have ~50% reduction in 5-HT reuptake compared to the long (L) allele [84], which may result in decreased 5-HT neurotransmission [85]. Reduced 5-HT availability leads to lower glucocorticoid receptor expression, which results in poorer negative feedback and greater cortisol secretion [51,52,53,54,55,56,57]. Stress hormones modify BDNF expression, such that there is decreased BDNF expression in the hippocampus and increased expression in the amygdala [94,95,96]. BDNF is intrinsic to neurogenesis and synaptogenesis [93], therefore, BDNF availability may contribute to the patterns of neuroconnectivity that develop as pain becomes comorbid and chronic [97,98]. Prolonged exposure to pain leads to a dampening of cortisol responses [52,53,66,67,68,69,70]. Inhibition of the HPA axis leads to heightened inflammatory/immune responses [203,204]. Therefore, following exposure to pain, large inflammatory responses in both the periphery and central systems may cause a reduction in neurogenesis and an increase in excitoxicity leading to both the chronification of pain and its comorbidities. Furthermore, glucocorticoids are involved in normal brain maturation and cell survival [205,206,207], and both an excess of or too low levels of cortisol can have deleterious effects on the brain, leading to long-term changes in behavior. HPA activity is programmed at birth [48,49], modified by the environment (e.g., parent behavior) [51,52,53,54,55,56,57,72], has significant effects in the developing brain [52,53], and influences responses to pain and behavior [63,64,65]. Pain, anxiety, depression and PTSD are all stress-related conditions influenced by either hyper- or hypo-glucocorticoid activity [63,64,65]. Evidence suggests that the combination of predisposing factors, along with the timing and duration of environmental influences, modifies HPA activity, which has widespread effects on the neurobiological (e.g., brain organization/function), physiological (e.g., arousal), and psychological systems (e.g., stress, anxiety sensitivity), and may lead to the maintenance of these conditions.

Precedence of mental health disorders onset prior to chronic pain have been reported [24,30]; however, the studies examining temporal relationships between the conditions are scarce, conflicting [208], and come with methodological limitations (e.g., reliance on retrospective report). Clinician-administered interviews (as opposed to self- and parental report) to prospectively assess mental and physical health in childhood, adolescence and early to middle adulthood would be a methodological advance that would help determine temporal relationships between chronic pain and psychiatric disorders.

When examining mental health and chronic pain in youth, it is crucial to take into account the parent–child relational context and the influential role of parental factors. Indeed, parents of youth with chronic pain report more anxiety, depression, and somatization symptoms than pain-free peers [167,173]. Moreover, maternal anxiety and depressive disorders were associated with depressive and somatic symptoms of children with chronic pain [167,172]. Therefore, it is possible that parents with mental health disorders through interactions with their genes and environment influence the comorbidity of chronic pain and mental health conditions in the child. It is necessary to further investigate the role of parental factors in the development and maintenance of pediatric chronic pain and comorbid mental health disorders and symptoms. Additionally, there is a paucity of research examining mental health in fathers of youth with chronic pain. Given the profound influence that pediatric chronic pain has on the entire family, it will be important to address paternal factors in addition to, and in combination with, maternal contributions to pediatric chronic pain and mental health conditions.

While psychological treatments for chronic pain have led to some changes in pain and disability, there is currently no evidence to suggest that it leads to lasting reductions in mental health symptoms. Moreover, many randomized controlled trials of psychological interventions for pediatric chronic pain have not included (or reported on) measures of PTSD, anxiety and depressive disorders over the course of treatment. These mental health conditions are often comorbid with one another, and many of the proposed mechanisms (e.g., avoidance, cognitive biases, sleep disturbances) underlying them are similar. Research in this area has tended to focus on these conditions and symptoms in isolation. Future research examining all of these mental health symptoms in the context of pediatric chronic pain is needed. Moreover, despite increasing evidence demonstrating that these comorbidities are common and debilitating, there is currently minimal empirically based guidance for how to treat children and adolescents who present with comorbid chronic pain and mental health conditions, and clinicians often rely on adult recommendations and/or clinical intuition. Clearly, treatment development and research on comorbid mental health conditions and chronic pain in youth is critically needed at this time. New therapies, which directly target the underlying neural mechanisms (e.g., rTMS; targeted anti-inflammatories) may also be a promising new direction for clinical intervention. However, whether or not these new treatments will be effective for treating chronic pain together with comorbid mental health disorders will need to be determined. Given potential intergenerational transmission of chronic pain and high levels of mental health symptoms among parents, research is needed to determine how to best address parents’ emotional burden. Novel parenting interventions to reduce parent distress in the context of pediatric chronic pain have been developed and tested and show promising evidence for improving parent and child emotional outcomes [180]. Addressing parent mental health conditions is likely a necessary first step before they can engage in behavior change that is often required in psychological treatments for their child’s chronic pain.

Sex differences in 5-HT signaling systems, stress responses, and neuroimmune interactions are being increasingly recognized [209,210]. Estrogens are often regarded as a central nervous system stimulant, and androgen receptor-mediated actions are often related to inhibition, which may underlie the lower incidence of many forms of chronic pain in males [211]. However, given the scope of this narrative review, in which we examined developmental trajectories of potentially shared neurobiological mechanisms underlying pain, PTSD, anxiety and depressive disorders, we were not able to describe the influence of sex hormones on each of these conditions, and how this changes across development. This is an important area for future research.

Given the debilitating impact of comorbid chronic pain and mental health disorders on youth there is an increasing need for prospective, interdisciplinary studies to identify the primary mechanisms underlying this co-occurrence in youth. This will inform the development and refinement of evidence-based interventions that can interrupt or possibly prevent the maintenance of these chronic conditions. Developmentally sensitive research integrating biological, behavioral, and social perspectives will be critical in driving forward this field of inquiry and addressing this growing epidemic.

## Figures and Tables

**Table 1:** Summary of epidemiological studies of internalizing mental health issues in youth with chronic pain.

Study	*n*	Design	Age (Year)	Assessment Time Points	Pain assessment	Internalizing Disorders/Symptoms Assessment	Findings
**Longitudinal studies of pediatric chronic pain and internalizing mental health issues into adulthood**							
Egger et al, 1998 [14]	1013	Longitudinal cohort study	5–15	Assessed annually over 3 years	CAPA somatization section	CAPA	40.8% of girls with depression reported headaches as compared to girls without depression (10.5%). 34.1% of girls with an anxiety disorder reported having headaches as compared with girls without an anxiety disorder (10%). 19.2% of boys with CD and 10% of boys with ODD reported having headaches as compared to boys without externalizing disorders (9.6%).
Egger et al, 1999 [15]	3733	Longitudinal cohort study	9–16	Assessed annually over 3 years	CAPA somatization section	CAPA	Girls with stomach aches (OR 7.2, CI 2.8–18.5) and musculoskeletal pain (OR 3.4, CI 1.5–8.0) were more likely to have anxiety as compared to pain-free girls. Boys with stomach aches were likely to have ODD (OR 3.6, CI 1.6–8.1) and ADHD (OR 3.5, CI 1.8–7.1) as compared to boys without stomach aches. Both girls (OR 12.9, CI 4.5–37.0) and boys (OR 10.5, CI 2.3–48.0) with musculoskeletal pain were more likely to report depression compared to children without musculoskeletal pain.
Hotopf et al, 1998 [5]	3637	Longitudinal cohort study	7–36	7, 11, 15 years old	Parent report of abdominal pain at ages 7, 11 and 15 years	N/A	Youth who had abdominal pain were more likely to develop a psychiatric disorder by Time 2 (OR 2.72, CI 1.65–4.49). Pain in childhood was not associated with heightened risk of physical symptoms in adulthood (OR 1.39, CI 0.83–2.36).
				36 years old	Self-report of physical symptoms (back pain, headache, abdominal pain, chest pain, dizziness, and rheumatism)	Semi–structured psychiatric interview (Present State Examination)	
Fearon et al, 2001 [3]	11,407	Longitudinal cohort study	7–33	7, 11, 16, 23 years old	Parent report of headache at ages 7 and 11 (binary variable)	Bristol Social Adjustment Guide	Youth suffering from frequent headaches were more likely to have recurrent headaches in adulthood (OR 2.22, CI 1.62–3.06), physical symptoms (OR 1.75, CI 1.46–2.10), and psychiatric disorders (OR 1.41, CI 1.20–1.66).
				33 years old	Self-report of physical symptoms (back pain, headache, twitches, rheumatism, indigestion, heart racing, worries about health)	Presence of four or more symptoms on a psychiatric morbidity self-report scale	
Walker et al, 2012 [16]	843	Longitudinal cohort study	12–21	12 years old	API, CSI	N/A	At 21 years, participants with High Pain Dysfunctional profile were at a higher risk of having a pain-related FGID (OR 3.45, CI 1.95–6.11), FGID and non-abdominal chronic pain (OR 2.6, CI 1.45–4.66), FGID and anxiety or depressive disorder (OR 2.84, CI 1.35–6.00) as compared with Low Pain Adaptive profile participants.
				21 years old	Rome III, PPQ	ADIS	
Shelby et al, 2013 [4]	491	Longitudinal cohort study	8–21	8–17 years old	Vanderbilt Pediatric Gastroenterology Service evaluation of FAP	N/A	At follow-up, participants with FAP were more likely to meet criteria for lifetime (OR 4.9, CI 2.83–7.43) and current (OR 3.57, CI 2.00–6.36) anxiety disorder and lifetime depressive disorder (OR 2.62, CI 1.56–4.40) as compared to controls. Participants with FAP, who developed FGID by follow-up, were more likely to meet criteria for any lifetime (OR 7.31, CI 4.17–12.81) or current (OR 5.09, CI 2.70–9.59) anxiety disorder and any lifetime depressive disorder (OR 4.14, CI 2.31–7.40) as compared to controls. Participants with FAP, who have not met criteria for FGID by follow-up, were still more likely to have any lifetime (OR 3.36, CI 2.01–5.63) or current (OR 2.68, CI 1.44–4.99) anxiety disorder as compared to controls.
				4 years after initial assessment	Rome III	ADIS	
Shanahan et al, 2015 [17]	1420	Longitudinal cohort study	9–26	9–16 years old, assessed 4–7 times	Self- and parent-report of recurrent (at least one one-hour episode at least once a week in the past three months) pain (headache, abdominal or muscle pain)	CAPA	34.4% of children reported somatic complaints. Participants with somatic complaints were more likely to have depressive (OR 6.90, CI 3.57–13.34) or anxiety (OR2.75, CI 1.55–4.89) disorders in childhood versus pain-free peers. Children with somatic complaints in childhood were more likely to develop depressive (OR 3.21, CI 1.54–6.70) or any anxiety disorders (CI 2.32, CI 1.30–4.14) by young adulthood as compared to pain-free participants. Sex differences in the likelihood of developing psychiatric disorders in adulthood following somatic complaints in childhood were not revealed.
				19, 21, 24–26	Recurrent headache binomial variable within the YAPA	YAPA	
Noel et al, 2016 [18]	14,790	Longitudinal cohort study	12–32	Wave I and II: 12–18	Self-report general health survey—frequency of headache, stomach ache, muscle/joints pain. Chronic pain was defined as pain at wave I and/or wave II	N/A	21.9% of participants reported having chronic pain during adolescence. Youth with chronic pain reported higher rates of lifetime depressive (24.5%) and anxiety (21.1%) disorders versus youth without chronic pain. Chronic pain in youth was associated with a greater likelihood of having lifetime anxiety (OR 1.33, CI 1.09–1.63) and depressive (OR 1.38, CI 1.16–1.64) disorders.
				Wave IV: ages 24–32	N/A	Diagnosis of PTSD, anxiety, and/or depression by a health care provider	
**Studies of co-occurring pediatric chronic pain and internalizing mental health issues**							
Balottin et al, 2013 [19]	1124	Meta-analysis	Mean ages: 11.6 (migraine), 12.3 (tension-type headache), 11.75 controls	N/A	ICHD I or II	CBCL	Having tension-type headaches was associated with higher internalizing symptoms (Hedge’s *g* = 2.344).
Blaauw et al, 2014 [20]	4872	Cross-sectional study	12–17	N/A	Headache interview assessing frequency of migraine, tension-type headache or unclassifiable headache over the last year	SCL-5	Recurrent headache of any type (migraine, tension-type) was associated with anxiety and depression symptoms (at the age of 12–14 years, OR 2.50, CI 1.61–2.61; at the age of 15–17 years, OR 1.64, CI 1.39–1.93).
Coffelt et al, 2013 [21]	3752	Retrospective cohort study	Mean age 13.54	N/A	Hospital record of chronic pain diagnoses (e.g., psychogenic pain not otherwise specified, chronic pain syndrome, complex regional pain syndrome)	Hospital record of a psychiatric diagnosis.	44% of youth with chronic pain have been diagnosed with a psychiatric condition, specifically, an affective (28%), anxiety (18%), somatization (6%) disorder or PTSD (2.4%).
Noel et al, 2016 [22]	195	Cross-sectional study	10–17	N/A	Self-report of pain characteristics (i.e., pain intensity, frequency, location, unpleasantness, and duration over the previous seven days); pain interference sub-scale of the PROMIS-25 Pediatric Profile	CPSS-5	32% of youth with chronic pain reported clinically significant PTSD symptoms as compared to 8% of pain-free peers. Parents of youth with chronic pain had higher levels of clinically significant PTSD symptoms (8%) as compared with parents’ of youth without chronic pain (1%). Among the chronic pain group, PTSD symptoms were significantly associated with pain intensity, unpleasantness, interference, and quality of life.
Simons et al, 2012 [23]	655	Retrospective chart review	8–17	N/A	11-point NRS	RCMAS	11% of youth with chronic pain reported clinically significant levels of anxiety, 31% underreported their anxiety levels.
Tegethoff et al, 2015 [24]	6483	Cross-sectional study	13–18 years	N/A	Self-report chronic pain conditions checklist	CIDI; parent-report SAQ	25.93% of youth reported having chronic pain and mental health disorder in their lifetime. Any type or chronic pain increased the risk of developing eating (OR 2.63, CI 1.63–4.24), anxiety (OR 2.42, CI 2.03–2.88), affective (OR 2.32, CI 1.85–2.91), or any mental (OR 2.51, CI 2.12–2.98) disorder. The onset of any mental health disorder preceded any chronic pain (OR 1.64, CI 1.44–1.86).

ADHD: attention-deficit/hyperactivity disorder; ADIS: Anxiety Disorders Interview Schedule-IV, Adult Lifetime and Child and Parent Versions; API: Abdominal pain index; CAPA: Child and Adolescent Psychiatric Assessment; CBCL: Child Behavior Checklist; CD: conduct disorder; CI: confidence interval; CIDI: Composite International Diagnostic interview; CPSS-5: Child PTSD Symptom Scale; CSI: Children’s Somatization Inventory; FAP: functional abdominal pain; FGID: functional gastrointestinal disorders; ICHD: International Classification of Headache Disorders; NRS: Numerical Rating Scale; ODD: oppositional defiant disorder; OR: odds ratio; PPQ: Persistent Pain Questionnaire; PROMIS-25: Patient-Reported Outcomes Measurement Information System; PTSD: posttraumatic stress disorder; Rome III: diagnostic questionnaire for functional gastrointestinal disorders; RCMAS: Revised Children’s Manifest Anxiety Scale; SAQ: self-administered questionnaire; SCL-5: Symptom Checklist; YAPA: Young Adult Psychiatric Assessment.
